# A scoping review of music-based interventions for swallowing difficulties: implications for treating older adults with presbyphagia

**DOI:** 10.3389/fmed.2023.1285835

**Published:** 2023-11-16

**Authors:** Soo Ji Kim, Myung Sun Yeo, So Yeon Kim, Seo Yeon Kang

**Affiliations:** ^1^Music Therapy Education, Graduate School of Education, Ewha Womans University, Seoul, Republic of Korea; ^2^Department of Music Therapy, Graduate School, Ewha Womans University, Seoul, Republic of Korea

**Keywords:** swallowing, music intervention, singing, scoping review, older adults, presbyphagia

## Abstract

**Objectives:**

Presbyphagia refers to age-related changes in the swallowing mechanism (e.g., reduced skeletal muscle strength that decreases bolus control). If left untreated, these changes can lead to dysphagia, which refers to impaired swallowing (e.g., coughing or choking when eating). Given that swallowing difficulties are common among older adults that they make up the fastest growing age group globally, the need for interventions to address presbyphagia is gaining urgency. To begin to address this need, we conducted a scoping review to analyze music therapy research aimed at enhancing swallowing function. The objective was to identify key intervention characteristics and propose clinical implications for treating presbyphagia using music therapy.

**Methods:**

This review followed the methodological frameworks outlined by Arksey and O’Malley and Levac et al. and used the Preferred Reporting Items for Systematic Reviews and Meta-Analysis for Scoping Reviews for analysis and reporting. Four electronic databases (i.e., ProQuest, PubMed, RISS, Web of Science) were searched for quantitative and qualitative studies in English or Korean that used music-based interventions to address swallowing function in older adults. Content analysis was conducted to identify and compare the main features of music interventions for swallowing difficulties among older adults.

**Results:**

Ten articles were identified and analyzed. It was found that three core components–respiration, vocalization, and singing–were employed to enhance swallowing function in populations with neurological impairments, dementia, or head and neck cancer. Notably, actions closely linked to swallowing function, such as laryngeal elevation and oral movements, were utilized therapeutically to speak or sing. Based on these characteristics, clinical implications are proposed to address presbyphagia.

**Conclusion:**

Singing entails a systematic and focused incorporation of stepwise activities that can be used to address swallowing disorders. In this context, critical clinical implications that music therapists should consider when treating individuals with presbyphagia include warmup breathing, vocalizing targeting laryngeal control, and singing targeting oral motor control. This review can contribute to the expansion of music therapy with older adults and the advancement of music therapy techniques.

## 1. Introduction

The global population of individuals aged 65 years or older currently stands at 720 million, with projections indicating a doubling of this figure within the next three decades (1). Among older adults, a prominent characteristic is the gradual decline in multiple physical functions, rendering them more vulnerable to conditions such as sarcopenia, dysphagia, osteoporosis, and frailty (2, 3). Given the rise in life expectancy and the multifaceted nature of aging, encompassing biological, psychological, and social changes, it is imperative we gain a deeper comprehension of how to effectively treat common age-related difficulties and disorders. One such difficulty is presbyphagia.

Aging leads to various changes in swallowing physiology, and these changes are collectively referred to as presbyphagia (4). While presbyphagia refers to normal age-related changes in the swallowing mechanism, these changes may be manageable if treated before they cause difficulties or serious dysfunction in swallowing. Difficulty swallowing is referred to by the umbrella term dysphagia and can be caused by neurological impairment, structural abnormalities, or muscle or brain disorders. Dysphagia has been found to affect over 20% of individuals over the age of 50 years in community settings and nearly 60% of individuals over the age of 80 years in nursing home settings (5).

Swallowing difficulties encompass both physiological and functional aspects, impacting not only psychological and social wellbeing but also overall health and communication (6, 7). As such, difficulties in swallowing can impact an individual’s quality of life. Notably, depression emerges as a significant symptom in this context, drawing attention in several studies (8, 9). These studies underscore a substantial link between swallowing difficulties and depression, especially among older adults. This emphasizes the significance of integrating psychological interventions with therapeutic approaches to effectively manage swallowing difficulties.

Swallowing is a neuroanatomical process composed of three stages: oral, oropharyngeal, and esophageal (10, 11). Specific structures such as muscles, nerves, the tongue, salivary glands, epiglottis, vocal cords, larynx, and hyoid bone are involved in each stage (11). Difficulties can arise from either mechanical obstruction or impaired motor function along the pathway through which food and liquid travel. Swallowing difficulties arising from neurological or structural causes necessitate rehabilitation interventions focused on precise motor timing and repetitive tasks to enhance function (12, 13). Interventions addressing swallowing difficulties aim to enhance laryngeal elevation and strengthen the associated muscles, ultimately improving swallowing function in dysphagic patients (14, 15).

The age-related changes defining of presbyphagia, such as diminished tongue pressure from muscle loss and extended upper esophageal sphincter opening times, increase an individual’s risk for dysphagia, including increased risk of aspiration (16). Unfortunately, conventional swallowing treatment modalities such as supraglottic swallow and Medelsohn maneuver, may be inappropriate or less effective with older populations (17). For instance, older adults commonly experience heightened fatigue and comorbidities that prohibit them from adhering the treatment. Therefore, when addressing swallowing changes and difficulties in older adults, it is crucial to consider the specific needs and limitations of this population, including the use of proper sensory activation and easily manageable swallowing motor tasks.

As an alternative to conventional treatment modalities, music-based interventions can target the oral and vocal structures involved in the process of swallowing and can be designed to meet the unique needs of older patients. In particular, singing can directly induce functional changes (18, 19). The basis for applying singing to improve swallowing function stems from respiration, phonation, and articulation sharing common neuroanatomical processes. Specifically, singing activates an auditory-motor feedback loop in the brain, which is further strengthened by the common neural network shared between singing and speaking (20). The processes of swallowing and vocalization are intricate coordination systems that necessitate precise integration of musculature in the upper airway, encompassing the oral, pharyngeal, laryngeal, and respiratory regions (21). Because singing requires coordination of respiration, vocalization, and articulation (22–24), it can address the needs of patients with dysphagia by integrating musical elements such as melody and rhythm. Thus, perceiving vocal melodies and integrating vocal and auditory information in speech production during singing involve neural engagement in auditory, linguistic, and emotional processing (25). The use of singing as a therapeutic method has been proven effective in the rehabilitation of diverse medical conditions, including stroke, aphasia, and mood disorders (26).

Singing has emerged as a promising rehabilitative approach in the realm of swallowing treatment, as evidenced by studies in neurorehabilitation (27, 28). While these singing-based music therapy approaches for dysphagia hold promise, the majority of the literature focuses on speech function (29, 30). It has been discovered that laryngeal elevation-based techniques applied in singing demonstrate favorable outcomes for patients with neurological impairment. The utilization of singing as a rehabilitative approach for improving swallowing function can be rationalized based on the evidence that the mechanisms involved in singing, including breathing, vocalization, and articulation (31), also play a crucial role in the process of swallowing.

Considering the need to expand the applications of music-based interventions, it is important to understand the theoretical and clinical characteristics of music-based intervention tasks. To address this need, we conducted a scoping review of music-based interventions focused on improving swallowing function. Scoping reviews use structured guidelines to synthesize information (32). This scoping review sought to gain insight from the research exploring the role of music-based interventions in swallowing rehabilitation and determine if there was consensus regarding the therapeutic components used to improve particular functional outcomes. Based on the analysis of the research, we suggest clinical implications for music therapists working with individuals with presbyphagia.

## 2. Methods

Levac et al. (33) refined the stepwise process originally outlined by Arksey and O’Malley (34) for scoping literature reviews, and we followed Levac et al.’s five stages, with the literature search and selection process being conducted iteratively. Additionally the research process, if there were areas that needed further supplementation based on data collection and review, continuous modifications were made to the process. In this study, we used the population, concept, context (PCC) framework to enhance the validity of the research presented in the JBI reviewer’s manual and other guidelines (35, 36). The framework’s detailed standards for classifying and analyzing information are the following;

### 2.1. identifying the research question(s)

The scope of the research on this topic is extensive, so questions regarding the research should use precise descriptions and specific inclusion criteria (33, 37). In other words, research questions must be defined clearly with criteria, as they provide a roadmap for subsequent stages, including research subjects, interventions, and outcomes. Therefore, this study constructed research questions considering the PCC framework. The PCC framework was used to establish the following:

- Population: Adults who experienced swallowing difficulties or swallowing disorders.
- Concept: The use of music-based interventions to treat swallowing difficulties/disorders among adults.
- Context: Publications describing music-based interventions for swallowing issues as a primary concern, written in either Korean or English.

Based on the PCC framework described above, the research questions for this study were as follows:

- What types of music-based intervention studies have been conducted to date that have addressed swallowing function in adults?
- What are the components of music-based interventions to improve swallowing function among adults?
- Given the components of music-based interventions for improving swallowing function, what are the clinical implictions for treating individuals with presbyphagia?

### 2.2. Searching the literature

A two-step search method was employed using electronic databases to conduct a literature search for published research. This method involved an initial search followed by reference and related literature searching. The procedures for selecting the database and keywords for the initial search were as follows.

#### 2.2.1. Selection of databases

Given the need for expertise and objectivity in music therapy interventions, we only reviewed published work from academic research databases. Conducting a literature review within the scope of the present topic requires collecting a substantial number of research findings. It takes time to comprehensively gather research results through keyword searches, especially for topics in which non-standardized terms are used interchangeably, such as in music therapy. In addition, the authors communicated with experienced researchers in the field of literature reviews and systematic literature analysis to ensure databases that offered advanced search capabilities and were commonly used for literature reviews were selected. Consequently, ProQuest, PubMed, Research Information Sharing Service, and Web of Science databases were used with no date restriction.

#### 2.2.2. Selection of search terms

The literature regarding music-based interventions for improving swallowing function was identified and analyzed. Music-based interventions use music to achieve therapeutic goals and may be implemented by music therapists or other professionals (38). Keywords commonly used in the field were supplemented with subject terms that aligned with the objectives of the study. To ensure a comprehensive search was conducted, the researchers modified the search terms and the sequence of database usage, repeating the search on different days to compare results. MeSH terms related to diagnoses (e.g., “swallowing disorder”) and musical techniques (e.g., “music,” “singing,” “vocal”) were combined with “intervention” and “therapy.” In addition, intervention-related terms such as “treatment,” “rehabilitation,” “training,” and “intervention” were employed, either individually or within specific categories in English and Korean. Research that was published in Korean was translated into English. The validity of the translation was verified through mutual discussion among the researchers. Table 1 shows the databases and search strategies used.

**Table 1:** Databases and search strategies using keywords.

Database	Search strategies and keywords
ProQuest (PsysARTICLES)	(“dysphagia” OR “swallowing” OR “deglutition” OR “swallowing disorder*”) AND (“music” OR “singing” OR “vocal task” OR “therapeutic singing”) AND (“intervention” OR “treatment” OR “rehabilitation” OR “training”)
ProQuest (PsyscINFO)	(“dysphagia” OR “swallowing” OR “deglutition” OR “swallowing disorder*”) AND (“music” OR “singing” OR “vocal task” OR “therapeutic singing”) AND (“intervention” OR “treatment” OR “rehabilitation” OR “training”)
PubMed	(“dysphagia” OR “swallowing” OR “deglutition” OR “swallowing disorder*”) AND (“music” OR “singing” OR “vocal task” OR “therapeutic singing”) AND (“intervention” OR “treatment” OR “rehabilitation” OR “training”)
Web of Science	(((TS = (dysphagia) OR TS = (swallowing) OR TS = (swallowing disorder*) OR TS = (deglutition))) AND ((TS = (music) OR TS = (singing) OR TS = (vocal task) OR TS = (therapeutic singing)) AND (TS = (intervention) OR TS = (treatment) OR TS = (rehabilitation) OR TS = (training))))
RISS	Each word in Korean: (((dysphagia) OR (swallow) OR (swallowing disorder)) AND ((music) OR (singing) (vocal task)) AND ((intervention) OR (treatment) OR (rehabilitation) OR (training)))

### 2.3. Stage 3: identifying studies

Inclusion criteria were the following: (a) implementation of a music-based intervention targeting swallowing function, (b) publication in English or Korean, and (c) reporting of measurable functional changes of swallowing or laryngeal diadochokinesis as a secondary assessment indicator of swallowing function. Exclusion criteria were (a) participants under 20 years of age, (b) duplicate intervention with the same participants, and (c) music listening intervention only. To remove duplicates and irrelevant articles, two researchers (SKi and SKa) screened article abstracts based on the previously described search strategy. Then the two other researchers (SJK and MY) revalidated the identified articles.

The assessment of study eligibility during the initial full-text screening involved two researchers (SJK and MY). Subsequently, to ensure the inclusion of studies aligned with the review’s objectives, the researchers conducted multiple rounds of full-text reviews. Instances of disagreement were resolved through consensus achieved during meetings and discussions. Final selection of articles was based on consensus.

### 2.4. Charting the data

The data retrieval and organization were done using EndNote X9 software from Clarivate Analytics (PA, USA). Then, to code the data, our framework was established in advance. Our framework includes general study information and details about the research questions. The charting table was completed independently by one researcher (MY) and peer reviewed by two researchers (SJK and SKi). A data extraction template was created to organize information from the identified articles. To ensure comprehensive data coverage, the researchers conducted the literature collection and selection processes twice, altering the order of the databases and sequence of the search terms.

To finalize the literature search, we used a modified data extraction form developed by JBI researchers (39). Two authors independently reviewed and cross-checked each selected literature and coded the data using Microsoft Excel 2022. The extracted data involved author name(s), publication year, country where the research was conducted, research methodology, participant population, intervention activities and their specific elements, reported outcomes, effects of the intervention, and details about the musical tasks used. We also categorized the intervention content described in each study by specific activity and analyzed the results to compare commonalities and differences across the studies. In addition, data collected about the music therapy interventions included intervention provider, intervention contents, intensity of sessions, presence of protocol groundwork, composition of musical tasks, and therapeutic rationale. Similar activities were grouped under the same category and classified accordingly.

The collected data was categorized and analyzed following the charting table’s detailed criteria (see Table 2).

**Table 2:** Charting framework’s detailed criteria.

Category	Sub-category	Details
General characteristics	Author information	Name, affiliation, academic field
	Publication-related information	Publication year/country, study design
	Participant information	Diagnosis, diagnosis of swallowing disorder, group assignment, number of participants, gender ratio of the intervention group/control group
Music-based intervention	Instructor	Music therapist or not
	Session information	Session time (min), intensity (per week), duration (weeks), total sessions, type (individual/group), accompaniment (live or recorded)
	Intervention details	Intervention steps and activities, music selection, therapeutic rationale, studies referenced in the protocol
Therapeutic goal	Measurement and outcomes	Assessment tools, outcome area, study outcomes

### 2.5. Collating, summarizing, and reporting the results

This stage of the scoping review was informed by the methodological framework outlined by Levac et al. (33). A comprehensive descriptive synthesis of the data presented in the charting table was conducted by three researchers (SJK, MY, SKi), while qualitative content analysis techniques were applied by two reviewers (SJK, MY) to formulate clinical strategies for addressing presbyphagia. The results from the synthesis and qualitative analysis were subsequently used to contextualize the results, specifically in relation to our research questions, and to inform the clinical implications surrounding the integration of music therapy sessions for individuals with presbyphagia. These insights also point to future research directions.

## 3. Results

**Figure 1:**
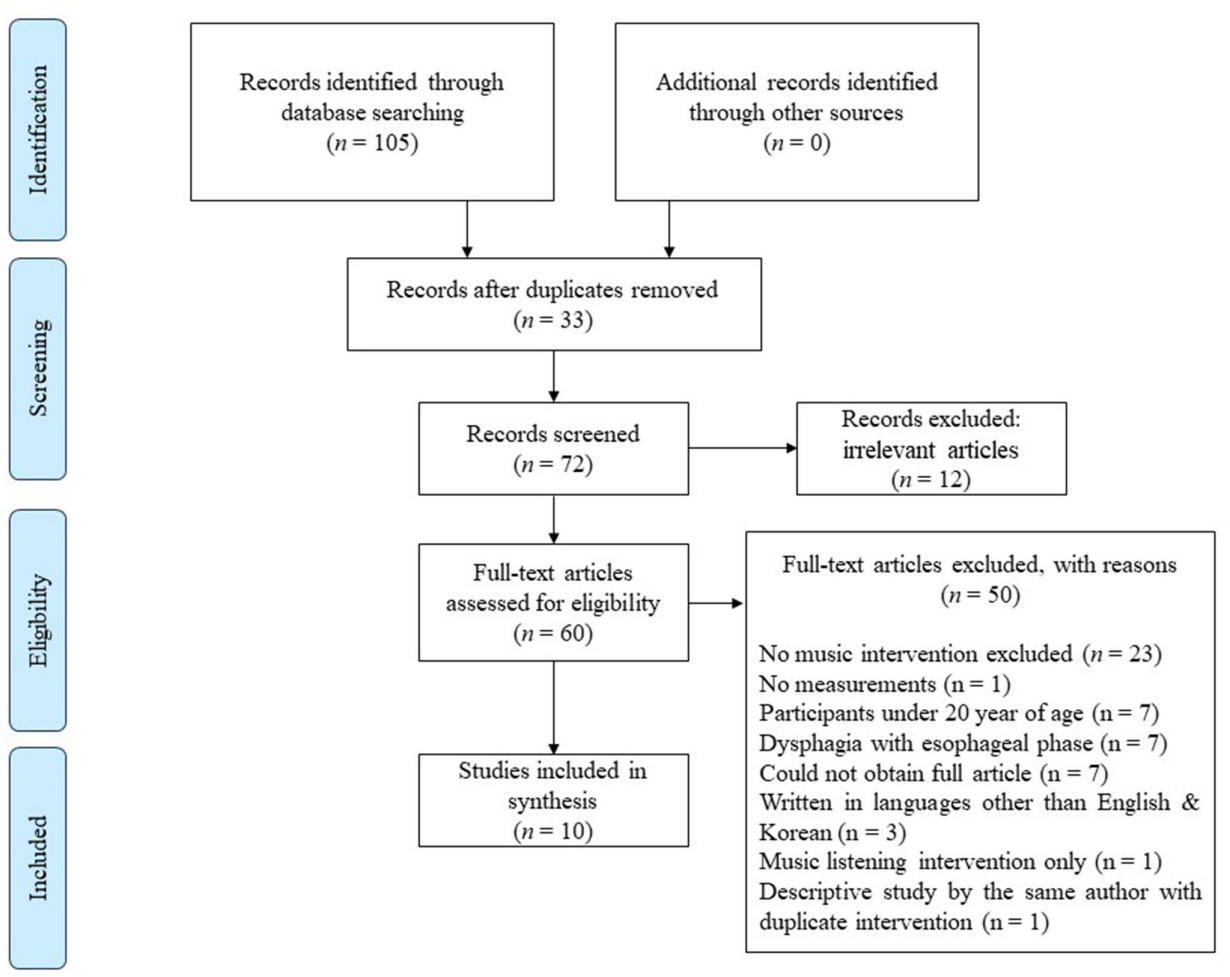
Workflow of the scoping review.

The search process identified 105 studies from the target databases. After duplicates were removed, 72 citations underwent title and abstract screenings. Following this 62 studies were excluded because they did not meet the inclusion criteria. Consequently, 10 studies were analyzed for this scoping review (see Figure 1).

### 3.1. What types of music-based intervention studies have been conducted to date that have addressed swallowing function in adults?

What types of music-based intervention studies have been conducted to date that have addressed swallowing function in adults?

#### 3.1.1. Study characteristics

Of the 10 studies included for analysis, two were published in 2010 (27, 40), five between 2012 and 2018 (28, 41–44), two in 2021 (45, 46), and one in 2022 (47). In terms of research design, there were three case studies (28, 41, 46), two randomized controlled studies (42, 45), four one-group pre/post studies (27, 40, 43, 44), and one ABA-mixed method case series study (47). The studies were conducted in Korea (6), USA (2), Japan (1), and Russia (1) (Table 3).

**Table 3:** Study characteristics (*N* = 10).

Author (year), Country	Study design	Patient description		Instructor, mode, frequency, duration	Intervention (steps, program)	Measurement	Main outcome
		Diagnosis (*N*)	N, sex (M:F), age (mean or range)				
Jomori and Hoshiyama (2010) (40) Japan	One-group pre/post study pilot	CVA (6), brain injury (1), dementia (1), disuse syndrome (2)	10 (4:6) 77.6 ± 5.6	NR; Gr 40 min/once (pilot)	6 steps (1) Introduction of the song; (2) Rhythmic movement activity with instruments; (3) Singing; (4) Rhythmic activity, beating a drum to song; (5) Instrumental performance for cool down; (6) Ending song by the therapist	(1) The number of swallowing movements (per hour) by EMG	(1) Sig. differences in swallowing rate between before and during music therapy for all participants. (2) Sig. high correlation coefficient between the increase in the frequency of swallowing during music therapy and the swallowing rate before music therapy.
Kim (2010) (27) Korea	One-group pre/post study	Stroke	8 (8:0) 58.7, (47, 49) 51–70	MT; In 15 min/ 3 times a week, 4 weeks	6 steps (1) Warm up vocalization (2 min); (2) Singing (3 min); (3) Two-step breathing using diaphragmic control (2 min); (4) Three-step breathing for 10 repetitions (2 min) (5) Laryngeal elevation through vocalization (3 min); (6) Singing for closing (3 min)	(1) Frenchay Dysarthria Assessment: reflex, respiration, laryngeal categories	(1) Sig. differences for pitch in the laryngeal section between the initial and mid-evaluation. (2) Sig. differences for dribble or drool in the reflex section, respiration at rest in the respiration section, laryngeal pitch, and speech in the laryngeal section between the initial and final evaluation.
Yun and Lee (2012) (44) Korea	One-group pre/post study	Dysphagia risk	29 (0:29) 66.48 ± 4.93	NR; Gr 60 min/ once a week, 8 weeks	3 steps *Based on Jomori, and Hosihiyama (40); Kim (27) (1) Breathing (2 min); (2) Vocalization (3 min) (3) Singing (50 min)	(1) Functional dysphagia scale (2) Modified water swallowing test (3) Food test (4) Nutrition state: TSF, MAMC	(1) Sig. differences in items related to preparatory disorders, aspiration risk, and pharyngeal disorders as well as in the total score on the functional dysphagia scale. (2) Sig. imprv. of pre- and post-intervention scores in the water swallowing test and food swallowing test.
Segall (2017) (42) USA	RCT	Elderly	A: 10 (NR) 85.5 ± 4.47	MT; Gr 45 min/ twice a week, A: 4 weeks B: 8 weeks	5 steps (1) Posture instruction and alignment (5 min) (2) Diaphragmatic breathing (10 min); (3) Vocal warmups (10 min); (4) Singing preferred songs (15 min); (5) Closure (5 min)	(1) MSP (2) Voice data: intensity, MPT	(1) Sig. imprv. in each group’s assessment items. (2) Difficult to determine group differences given different intervention periods.
			B: 10 (NR) 85.5 ± 4.47				
Stegemöller et al. (2017) (43) USA	One-group pre/post study	IPD	HD: 6 (NR) 65 ± 11	MT; Gr NR/ HD: twice a week, 8 weeks LD: once a week, 8 weeks	2 steps (1) 4 series of vocal exercises (2) Group singing	(1) UPDRS (2) EMG (3) SWAL-QOL	(1) Sig. imprv. in EMG outcome measures. (2) Sig. imprv. in UPDRS total and UPDRS motor scores. 3) No sig. Differences in SWAL-QOL.
			LD: 6 (NR) 69 ± 7				
			LD: 6 (NR) 69 ± 7				
Jo et al. (2018) (41) Korea	Case study	Brain injured	1 (1:0) 82	NR; In 30 min/ 5 times a week, 4 weeks	2 steps (1) Humming, vocalization, singing with lyrics (15 min); (2) Wind instrument playing (15 min)	(1) MMSE-K (2) BASOFF (3) Spirometer (4) Modified dysphagia risk assessment scale	(1) Imprv. of cognitive functions. (2) Imprv. of oral motor function during meals. (3) Imprv. of respiratory function. (4) Imprv. (reduction) in dysphagia risk score.
Yeo and Kim (2018) (28) Korea	Case study	Tongue cancer (1), SCI (1), PD (1)	3 (2:1) 62.3 (50–78)	MT; In 30 min/ 2–3 times a week, about 4 weeks	4 steps (1) Respiratory muscle relaxation and warmup (2) Vocalization and therapeutic singing (3) Vocal training for laryngeal elevation (4) Breathing for conclusion	(1) Voice data: intensity, jitter, L-DDK, MPT, NHR, pitch (2) SWAL-QoL	(1) Imprv. of L-DDK, MPT, SWAL-QoL in all patients. (2) Imprv. of patients’ voice quality.
Jo et al. (2021) (45) Korea	RCT	HNC	Con:13 (10:3) 59.15 ± 4.22	MT; In 20 min/ 3 times a week, 4 weeks	4 steps (1) Physical preparation with breathing for relaxation (2 min); (2) Vocal warmup (3 min) (3) Singing for laryngeal elevation (10 min) (4) Modified singing (5 min)	(1) DIGEST (2) VDS (3) Voice data: intensity, jitter, L-DDK, MPT, NHR, pitch, shimmer	(1) Sig. imprv. of L-DDK in the intervention group compared to that of the control group. (2) Sig. imprv. of VDS, DIGEST in IG, and items in the pharyngeal phase score of VDS. (3) Sig. imprv. of the pharyngeal CG in VDS, DIGEST score.
			Inter:15 (9:6) 50.87 ± 3.68				
Yeo et al. (2021) (46) Korea	Case study	PD, HNC	4 (1:3) 65.8	MT; In 30 min/ twice a week, 12 weeks	5 steps (1) Respiratory muscle relaxation; (2) Vocal folds relaxation by vocalization; (3) Laryngeal elevation by vocalization of two vowels; (4) Modified singing for respiratory control; (5) Articulation based on musical phrases or accents	(1) VDS (2) VFSS (3) Voice data: AMR, L-DDK, MPT, SMR (4) SWAL-QoL	(1) Imprv. of L-DDK, SWAL-QoL in all patients. (2) Imprv. of VDS in all patients associated with different swallowing tasks for each patient group. (3) Decrease of MPT in PD, HNC, increase in PD.
Apreleva Kolomeytseva et al. (2022) (47) Russia	ABA-mixed method case series study	ALS	8 (2:6) 58.1	MT; In About 60 min/ twice a week, 6 weeks	15 steps (1) Opening and assessment (5 min); (2) Body alignment (3 min); (3) Diaphragmatic breathing (4 min); (4) Controlled breathing and lip seal (3 min); (5) Music assisted relaxation for voice (8 min); (6) “Ping pong” soft palate (1 min); (7) Phonation (5 min); (8) Consonant range cantillation (2 min); (9) Velopharyngeal port (3 min); (10) Impulse diaphragmatic breathing (2 min); (11) sustained vowels production (5 min); (12) Laryngeal elevation through vocalization (5 min); (13) Vocal cord relaxation (2 min); (14) Preferred song performance (5 min); (15) Closure and assessment (5 min)	(1) MIP, MEP, FVC (2) PEF; (3) VFSS (4) CNS-BFS swallowing, speech sub score (5) Voice data: MPT, AMR, SMR, jitter, shimmer, NHR, VSA (6) F0, speaking rate, speech pause ratio/ pause frequency during oral reading (7) F0, speaking rate, speech–pause ratio/ pause frequency during spontaneous speech	Imprv. of bulbar and respiratory functions as follows: (1) MIP, MEP (2) PEF (3) Hypernasality level, time-to-laryngeal vestibule closure, maximum pharyngeal constriction area, peak position of the hyoid bone, total pharyngeal residue C24 area (4) CNS-BFS swallowing and speech subscales (5) MPT, jitter, shimmer, NHR (6) Speaking rate, speech–pause ratio, pause frequency

ABA, applied behavior analysis; ALS, amyotrophic lateral sclerosis; AMR, alternating motion rate; BASOFF, Behavioral Assessment Scale of Oral Functions in Feeding; Con, control group; CNS-BFS, Center for Neurologic Study - Bulbar Function Scale; CVA, cerebral vascular disease; C24 area, area of cervical vertebrae C2 to C4; DIGEST, dynamic imaging grade of swallowing toxicity; EMG, Electromyography; F, female; FVC, forced vital capacity; F0, frequency; Gr, group intervention; HD, high dosage group; HNC, head and neck cancer; Imprv., improvement; In, individual intervention; Inter, intervention group; IPD, idiopathic Parkinson’s disease; L-DDK, laryngeal diadochokinesis; LD, low dosage group; M, male; MAMC, mid arm muscle circumference; MEP, maximal expiratory pressure; MIP, maximal inspiratory pressure; MMSE-K, mini mental state examination-k; MPT, maximum phonation time; MSP, mean swallowing pressure; MT, music therapist; N, no; NHR, mean noise to harmonics ratio; NR, not reported; PD, Parkinson’s disease; PEF, peak expiratory flow; RCT, randomized control trial; SMR, sequential motion rate; SCI, spinal cord injury; Sig., significant; SWAL-QOL, Swallowing Quality of Life questionnaire; TSF, triceps skin fold thickness; UPDRS, unified Parkinson’s disease rating scale; VDS, video fluoroscopic dysphagia scale; VFSS, video fluoroscopic swallowing study; VSA, vowel space area.

To reiterate, a diagnosis of a swallowing disorder was not required for inclusion in this review. Presbyphagia symptoms can occur without meeting the criteria for dysphagia. It was hoped that by identifying and analyzing music-based interventions focused more generally on swallowing difficulties in adults that insight into the treatment of presbyphagia could be obtained. Among the 10 studies, four applied singing interventions to improve swallowing disorders due to neurological impairments. One study included older adult participants without swallowing disorders (42) and another included older adult participants at-risk for dysphagia. Singing interventions were also applied to swallowing disorders due to head and neck cancer (HNC) (45). Two studies included mixed diagnoses with participants diagnosed with either cancer and brain damage (46) or stroke and dementia (40). It is important to note that the location of a tumor can impair swallowing as can radiation from cancer treatments. Brain damage from stroke or dementia can also interfere with one’s ability to swallow.

The number of participants who completed the intervention in each study varied from 1 to 29. In one of the three case studies, a single participant was involved (41), while the other two case studies each had three participants (27, 46). The ages of the participants in all 10 studies exceeded 50 years; three studies included participants aged 60 and above (43, 44, 46), and two studies had an average participant age of over 80 years (41, 42). Of the 10 studies reviewed, six studies employed individual intervention (27, 28, 41, 44–46), whereas four studies utilized group intervention (40, 42–44). In terms of conducing sessions, seven studies involved music therapists (27, 28, 41–43, 46, 47), while three did not report facilitators (40, 41, 44).

Regarding outcome measurements, vocal components, articulation, and swallowing functions were measured. Vocal aspects included measures like maximum phonation time (MPT) and vocal quality (jitter, shimmer, Noise-harmonics to ratio, NHR), while articulation involved tasks such as alternating motion rate (AMR) and sequential motion rate (SMR). For swallowing, direct measures like video fluoroscopic swallowing study (VFSS), video fluoroscopic dysphagia scale (VDS), Dynamic Imaging Grade of Swallowing Toxicity (DIGEST), modified water swallowing test, and mean swallowing pressure were utilized. Indirect methods encompassed assessments within the Frenchay dysarthria assessment related to swallowing functions, laryngeal diadochokinesis (L-DDK) test related to laryngeal-muscle movement, and electromyography (EMG), among others. Only three studies used the Swallowing Quality of Life (SWAL-QoL) questionnaire (28, 43, 46).

### 3.2. What are the components of music-based interventions to improve swallowing function among adults?

#### 3.2.1. Music intervention component analysis

Within the scope of the scrutinized literature, the therapeutic applications were grounded in singing, commonly employed for speech rehabilitation. As an extended and modified use of the speech production system, singing can be a key element of swallowing interventions in music therapy (48, 49). Upon examining the interventions used in each study, they generally applied musical components to focus on swallowing functions in the process of singing, encompassing the respiratory, vocalization, and articulatory stages. To identify the core components of music-based interventions targeting swallowing function, we reviewed not only the intervention contents but also all relevant description details within the studies as well as key reference materials.

The constituent activities within the structured music interventions were subjected to analysis (see Table 4). Examining the studies based on the executed activities, it became apparent that the predominant tasks were ‘upper body movement – stretching arms, neck, and shoulders to relax the muscles (28, 41–44, 46, 47), and ‘vocal sound gliding using a vowel sound’ (27, 28, 42–47), which was reported in 8 studies among 10 studies. Conversely, activities involving direct stimulation of the laryngeal muscles, pertinent to swallowing, were featured in only four studies (27, 28, 45, 46). The music tasks related to laryngeal movements closely involved in the swallowing process and airway protection were identified as ‘stepwise breathing’ (27) and ‘vocalizing (/ah/)-/oo/-/ee/ in ascending pitch order’ (27, 28, 45, 46). In addition to these, activities related to vocal musculature and orofacial motor skills included humming (27, 28, 41, 45, 46) and sound gliding (27, 28, 42–47), as well as vocalizing the vowel /a/ while exhaling after inhaling sufficiently (46, 47).

**Table 4:** Analysis of intervention activities based on goals, target function, approach, studies, and population.

Intervention activity	Target therapeutic goal	Included study	Suggested activities for presbyphagia
Diaphragmatic breathing	Relaxing respiratory muscles	*N* = 3: Segall (42), Stegemöller et al. (43), Apreleva Kolomeytseva et al. (47)	Body alignment for relaxation
Upper body movement – stretching arms, neck, and shoulders to relax the muscles	Relaxing respiratory muscles	*N* = 8: Yun and Lee (44), Segall (42), Stegemöller et al. (43), Jo et al. (41), Yeo and Kim (28), Jo et al. (45), Yeo et al. (46), Apreleva Kolomeytseva et al. (47)	Head and neck movement (head posture change)
Singing a short phrase of melodies with regular breathing between phrases	Enhancing targeted articulatory system	*N* = 1: Yeo et al. (46)	Respiratory muscle movement
Singing a preferred song in a comfortable tempo and pitch range	Inducing laryngeal muscle movement	*N* = 6: Segall (42), Stegemöller et al. (43), Jo et al. (41), Jo et al. (45), Yeo and Kim (28), Yeo et al. (46)	Respiratory muscle movement
Vocal sound gliding using a vowel sound	Relaxing vocal musculature	*N* = 8: Kim (27), Yun and Lee (44), Segall (42), Stegemöller et al. (43), Yeo and Kim (28), Jo et al. (45), Yeo et al. (46), Apreleva Kolomeytseva et al. (47)	Vocal warmup
Vocalizing the vowel /a/ while exhaling after inhaling sufficiently	Relaxing vocal musculature	*N* = 2: Yeo et al. (46), Apreleva Kolomeytseva et al. (47)	Vocal warmup
Humming	Relaxing vocal musculature	*N* = 5: Kim (27), Jo et al. (41), Jo et al. (45), Yeo and Kim (28), Yeo et al. (46)	Vocal warmup
Stepwise breathing	Inducing airway protection	*N* = 1: Kim (27)	Laryngeal muscle movement
Vocalizing (/ah/)-/oo/-/ee/ in ascending pitch order	Elevating larynx	*N* = 4: Kim (27), Yeo and Kim (28), Jo et al. (45), Yeo et al. (46)	Laryngeal elevation
Vocalizing using a consonant sound	Regulating orofacial musculature	*N* = 3: Jo et al. (41), Stegemöller et al. (43), Apreleva Kolomeytseva et al. (47)	Oral motor exercise
Singing a modified song repetition of target orofacial movement	Stimulating orofacial motor	*N* = 4: Jo et al. (41), Jo et al. (45), Yeo et al. (46), Apreleva Kolomeytseva et al. (47)	Oral motor exercise

Upon conducting a detailed analysis of each activity in accordance with the therapeutic goals for swallowing function, the following results were obtained. Relaxing of respiratory muscles (*n* = 2) was attempted in a form of diaphragmatic breathing and upper body stretching. Enhancing targeted articulatory system (*n* = 1), and inducing laryngeal muscle movement (*n* = 1) were attempted in a form of singing, while relaxing vocal musculature (*n* = 3) was achieved through activities such as vocal sound gliding, vocalizing vowel sounds, or humming. Activities that directly stimulate the larynx, which plays the most direct role in the swallowing process, include inducing airway protection (*n* = 1), elevating the larynx (*n* = 1), regulating orofacial musculature (*n* = 1), and stimulating orofacial motor skills (*n* = 1).

#### 3.2.2. Vocalization and singing for swallowing

Vocalization activities were undertaken in nine of the studies with diverse objectives and methods. The objectives of these vocalization activities encompassed soft palate elevation, vocal cord relaxation, and laryngeal elevation. In terms of methods, five studies (27, 28, 45–47) employed vocalization in the form of glissando or humming to facilitate vocal cord relaxation. These vocalization activities were subdivided into preparatory and execution stages. The preparatory stage of vocal activities typically involves comfortably and lengthily producing the vocalizing vowel /a/ or humming, while the execution stage corresponds to actively controlling vocal structures’ movements to produce sounds with varying pitch while singing.

Regarding intervention activities, several methods were utilized. These included brief glissando utterances by the participant, subsequently reflected upon by the therapist (27, 28, 45, 46), as well as modeling (42). Additionally, three studies incorporated aspects of adjusting pitch or tempo given by music therapist (27, 28, 45). Beyond the vocal preparation phase, the primary objectives extended to sustaining laryngeal elevation, enhancing voice intensity, and strengthening motor function in the oral muscles. Furthermore, the introduction of vocal training through diverse methods, including vocal instruction, glissando, and messa di voce (a vocal method that involves maintaining a consistent pitch while gradually increasing the volume of the voice), highlighted the goals and techniques that were utilized.

All studies incorporated a singing phase as part of their intervention. Preferred song singing was performed in two studies (28, 43), and in seven studies singing was carried out while modifying respiration or lyrics (27, 41, 42, 44–47). Only one study (40) included preferred song singing without specific therapeutic goals and described this as a rhythmic activity.

#### 3.2.3. Use of lyrics to focus on targeted oral motor movement

Among the studies involving singing with lyrics, five used singing to improve physical functions such as breathing and articulation (28, 41, 44–46). Activities using vocalization of vowels and consonants or singing with lyrics to strengthen the orofacial muscles were presented in all studies. Singing was performed by inserting consonants instead of lyrics. In a study with the primary goal of strengthening oral muscles, a detailed description of the spherical shape of vowels and rationale for the selection criteria of consonants to replace lyrics were presented. It was reported that vocalization using vowels induces various movements of the jaw and helps to strengthen the muscles of the lips, cheeks, and jaw. Also, Korean palatal sounds, such as /gah/ and /kah/, and Korean tongue consonants, such as /tah/, were used to maximize tongue movement.

The manner in which singing was conducted also exhibited variations depending on the type of intervention. In certain cases, singing activities within group interventions were intertwined with functions like articulation and breathing (43, 44), while in others, they pertained to speech function (28, 41–43, 45–47). Notably, these activities pursued goals not directly aligned with swallowing function. Additionally, interventions targeting swallowing function, both in individual and group settings, predominantly comprised breathing and vocalization exercises, as opposed to conventional singing.

#### 3.2.4. Enhanced musical experience

Regarding the use of music, six studies meticulously outlined the therapist’s accompaniment choices (27, 28, 43, 45–47) For instance, during breathing exercises, a trill accompaniment was introduced to signal breath holding during breathing activity or adjustment was made to accommodate the participant’s vocal range and voice intensity during singing. The music excerpt selected for the patient’s involvement was tailored according to the patient’s vocalization.

In contrast, the remaining four studies (40–42, 44) did not delve into the therapist’s musical involvement, as they refrained from specifying the musical excerpt employed by the therapist or the music incorporated. Instead, their focus rested on monitoring the activity’s progression. The singing process appeared to be centered around general singing without specific musical modifications for a particular purpose. However, the therapist’s musical proficiency emerged as a pivotal factor, as it served as an effective strategy for enhancing engagement and refining participant performance.

When considering vocalizations, the incorporation of musical components into singing activities stands out as crucial, employing an array of approaches. The therapist’s musical expertise assumes a central role in shaping this process, adjusting song composition through alterations in accompaniment and the integration of musical elements. While prioritizing singing activities that accentuate articulation, the prevalent approach involved the direct rendition of familiar songs devoid of a therapeutic rationale.

### 3.3. Research question 3: given the components of music-based interventions for improving swallowing function, what are the clinical implications for treating individuals with presbyphagia?

#### 3.3.1. Clinical implications of singing to treat presbyphagia

Given the results of this scoping review, we offer clinical implications for music therapy addressing age-related swallowing difficulties in older adults. Singing has clinical viability because it is not sensitive to the patient’s level of cognitive functioning. Considering the attributes of the aging demographic, the co-occurrence of cognitive deterioration is anticipated, thereby amplifying the clinical strength of singing as an intervention that holds significant accessibility for this specific cohort.

In addition, music therapy interventions for presbyphagia should place significant emphasis on respiration, vocalization, and singing. These facets involve extensive engagement and straightforward implementation for a broad segment of the older adult population. To effectively address presbyphagia, these music therapy elements should align with physical activity and target muscle strengthening pertinent to swallowing. Patients should be able to execute these tasks independently to maintain a consistent level of intervention intensity and experience the psychological benefits of music-related activity (49, 50).

In interventions incorporating singing, respiratory exercises conducted at the initial stage were predominantly employed alongside relaxing music to elevate activity levels or stretch associated muscles. However, when designing interventions that target swallowing function, particularly tailored for older adults, controlled respiratory exercises are essential, encompassing stronger dynamics and brief breath pauses for practicing the complete closure of the epiglottis, a structure related to swallowing. Hence, there is a need to cautiously incorporate sufficient repetition of regulated respiratory maneuvers.

Vocalization is a vital function closely tied to swallowing, with crucial laryngeal muscle control. Vocal folds, false vocal folds, and aryepiglottic folds contract in tandem with hyoid and laryngeal elevation, protecting the airway (51). Furthermore, oral control and lingual control are integral to vocalization and swallowing. Both non-speech vocalization and speech demand mouth opening and an upright head position for effective sound projection. Speech adds the complexity of coordinating lip, jaw, and tongue movements; shaping oral-pharyngeal cavities for vowel resonances; and forming obstructions for consonants, requiring synchronization with voice onset and offset (52).

**Figure 2:**
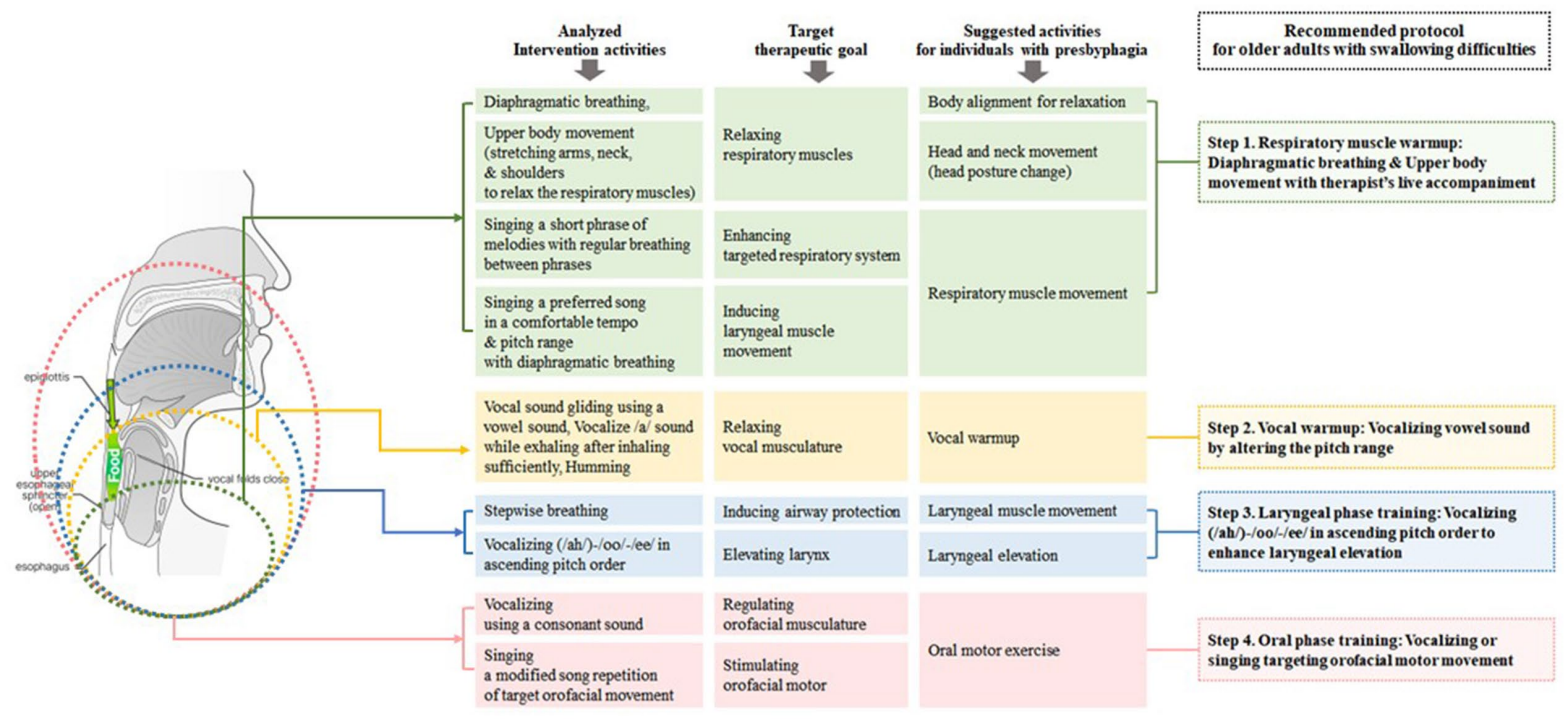
Proposed clinical implications based on research analysis.

Considering the relationship between vocalization and swallowing (53), it becomes apparent from the current research analysis that activities such as humming, vowel sound production, gliding vowel sounds, and vocalizing at different pitches have tended to center around the oral phase. In light of the age-related decline in muscle function among older individuals, movements necessitating resistance, such as laryngeal elevation, should be more proactively integrated. To this end, a diverse array of singing such as vocalizing or singing melodies with various pitch intervals can be effectively employed. Furthermore, avenues for facilitating more vigorous movements of structures like the tongue and jaw need to be explored in vocalization. In singing, careful consideration must be given to the choice of lyrics for vocalization exercises, specifically utilizing lyrics containing certain pronunciations and incorporating direct tongue protrusion and yawning. The overall clinical implications are summarized in Figure 2.

## 4. Discussion

The purpose of this article was to analyze relevant research to present the theoretical frameworks and intervention protocols using music-based interventions for swallowing difficulties in adults within rehabilitation settings. Considering the growing need to address swallowing difficulties among older adults, it is necessary to expand the scope of music therapy interventions by looking at the clinical applications of previous research findings. As such, this review synthesized the music therapy applications for swallowing interventions and analyzed components of musical tasks tailored to facilitate swallowing function. Not surprisingly, the majority of studies examined in this review included singing tasks encompassing various target subtasks, such as orofacial muscle exercises and vocalizations, which could be implemented by older adult populations.

Compared to a previous scoping review of singing-centered interventions in pulmonary rehabilitation, the current analysis showed that singing interventions for functional improvement were structured to include respiration, vocalization, and singing. Studies exploring singing in respiratory rehabilitation tend to prioritize community-based group singing, so there was less specific information regarding the therapeutic aspects of singing tasks for functional improvement (54). It implies that understanding therapeutic considerations in composing music therapy tasks for target behaviors is essential. This aligns with our findings, showing a consistent intervention structure. However, our review found specific vocal and singing tasks associated with swallowing function, suggesting a deliberate inclusion of aspects related to swallowing function.

Among the examined studies, seven (27, 28, 41, 42, 45–47) evaluated the relationship between oral and language functions in swallowing capabilities. Singing tasks are inherently tied to respiratory control, articulation, and phonation, all of which prominently impact structures involved in swallowing function. Changes observed in these parameters underscore the potential of a singing-based enhancement protocol to exert a comprehensive influence on individuals with presbyphagia. Studies that assessed alterations in laryngeal AMR and L-DDK before and after interventions consistently found proof that musical interventions possess the capacity to concurrently enhance both swallowing and speech functions (28, 45, 46). Furthermore, activities promoting movements of the jawbone or tongue, pivotal for precise articulation, share commonalities with the mechanisms inherent in singing, wherein the lyrical content is structured. Consequently, if forthcoming endeavors integrate singing activities focused on enhancing articulatory engagement, a mutual advancement in both the participants’ swallowing and speech functions could be reasonably anticipated.

In music therapy sessions, selecting and modifying lyrics tailored to each patient are significant tasks for music therapists. Generally, this involves considering mechanisms such as auditory-motor interactions (55). For swallowing function, there are differences in the treatment direction due to the high involvement of more complicated timing and the movement of oral and pharyngeal regions. In the interventions included in this scoping review, vocal activities like humming and gliding, which were predominantly employed, went beyond mere warmup exercises and served as direct goal-oriented activities manifested by lengthy duration and the inclusion of multiple tasks during the vocalization phase of the intervention [i.e., (43, 46)]. Thus, for older adults with decreased muscular strength, a more proactive and efficient implementation of music applications should be developed to induce functional improvement.

### 4.1. Strengths and limitations

Scoping reviews inherently have limitations in thoroughly exploring the information extracted from their findings, as their primary focus is to outline the extent of published works on a given topic. Consequently, this particular review refrained from systematically evaluating the quality of the included studies. In terms of understanding the research topic, all authors are qualified music therapists and researchers with clinical experience in music therapy interventions targeting swallowing function. Notably, this review is the first of its kind to examine music interventions aimed at improving swallowing function; however, due to the limited availability of relevant research, there are limitations in extrapolating the specifics of these interventions. Since not all included studies were conducted by music therapists, there may be variations in the composition and level of expertise in interventions. Given the lack of prior research on music therapy interventions for presbyphagia, this analysis included different music-based interventions designed to enhance swallowing function across various groups of participants. From this approach, clinical implications were derived.

## 5. Conclusion

This scoping review holds the potential to enhance the efficacy of music therapists in addressing age-related swallowing disorders prevalent among the aging population. This research provides valuable insights for music therapists to respond more effectively to the challenges posed by age-related swallowing difficulties. Given that singing, a frequently employed musical activity within music therapy, shares structural elements with the phonation process, its utility in augmenting speech function is well understood. While singing to treat swallowing difficulties appears to parallel its application for speech enhancement, its direct extension to improving swallowing function is limited.

In the context of ameliorating age-related swallowing difficulties, it is essential to structure music therapy interventions that encompass precise muscular control and heightened execution intensity of anatomical structures intrinsically linked to swallowing function. It is anticipated that music therapy in clinical settings will witness a growth in treatment tailored to the indispensable mechanism of swallowing, consequently contributing to an enhanced quality of life for the older adult population.

## Author contributions

SJK: Conceptualization, Data curation, Formal analysis, Investigation, Methodology, Writing – original draft, Writing – review & editing. MY: Conceptualization, Formal analysis, Validation, Writing – original draft, Writing – review & editing. SKi: Data curation, Formal analysis, Visualization, Writing – review & editing. SKa: Data curation, Validation, Visualization.
